# Microstructure and Mechanical Properties of Wire Laser Additive Manufactured Deposits and Their Tungsten Inert Gas Welds

**DOI:** 10.3390/ma18061308

**Published:** 2025-03-16

**Authors:** Yeong Rae Shim, Jong Kun Kim, Deok Hyun Jo, Hee Pyeong Yang, Seung Wook Yoon, Un Yong Yu, Hyub Lee, Durim Eo, Jong Cheon Yoon, Sunmi Shin, Joong Eun Jung, Jong Bae Jeon

**Affiliations:** 1 Department of Materials Science and Engineering, Dong-A University, Busan 49315, Republic of Korea; yrshim@kims.re.kr (Y.R.S.); kimjg@airkpc.com (J.K.K.); deoghyeonjo894@gmail.com (D.H.J.); heepyeong1@gmail.com (H.P.Y.); 2 Department of Extreme Materials Research Institute, Korea Institute of Materials Science, Changwon 51508, Republic of Korea; 3 Department of Innovation Support, KP Aero Industries Co., Ltd., Gimhae 50875, Republic of Korea; ysw@airkpc.com (S.W.Y.); durogo@airkpc.com (U.Y.Y.); 4 Korea Additive Manufacturing Innovation Center, Korea Institute of Industrial Technology, Siheung 15014, Republic of Korea; leehyub@kitech.re.kr (H.L.); adream@kitech.re.kr (D.E.); jongcheon89@kitech.re.kr (J.C.Y.); 5 Ulsan Technology Application Division, Korea Institute of Industrial Technology, Ulsan 44413, Republic of Korea; smshin@kitech.re.kr

**Keywords:** Ti-6Al-4V, wire laser additive manufacturing, tungsten inert gas welding, defect, microstructure

## Abstract

Ti-6Al-4V (Ti64) alloy is widely utilized in the aerospace industry due to its high strength, fatigue resistance, corrosion resistance, and cryogenic properties. However, its high raw material costs and machining difficulties necessitate the development of efficient manufacturing processes. This study evaluates the mechanical reliability and microstructure of Ti64 components fabricated using wire laser additive manufacturing (WLAM) and subsequently joined via tungsten inert gas (TIG) welding. The WLAM process produces refined microstructures with superior mechanical properties by minimizing defects; however, insufficient process optimization may result in a lack of fusion (LOF) and porosity. Microstructural analysis revealed that the WLAM deposits exhibited a fine basket-weave α structure with an average α-lath width of 1.27 ± 0.69 μm, while the TIG-welded region exhibited a coarsened α-lath, reaching 3.02 ± 2.06 μm, which led to a reduction in ductility. Tensile testing demonstrated that the WLAM deposits exhibited superior mechanical properties, with a yield strength of 910 MPa, ultimate tensile strength of 1015 MPa, and elongation of 12.8%, outperforming conventional wrought Ti64 alloys. Conversely, the TIG-welded joints exhibited reduced mechanical properties, with a yield strength of 812 MPa, ultimate tensile strength of 917 MPa, and elongation of 7.5%, primarily attributed to microstructural coarsening in the weld region. The findings of this study confirm that WLAM enhances the mechanical properties of Ti64, whereas TIG welding may introduce structural weaknesses. This research provides insight into the microstructural evolution and mechanical behavior of WLAM-fabricated Ti64 components, with valuable implications for their application in aerospace structures.

## 1. Introduction

Ti-6Al-4V (Ti64) alloy occupies approximately 50% of the titanium alloy market and is extensively applied in the aerospace industry due to its high tensile strength, exceeding 900 MPa; excellent fatigue resistance, capable of withstanding 10^9^ cycles at stress levels above 400 MPa, and outstanding cryogenic properties, including an ultimate tensile strength of 1298 MPa and an elongation of 12.5% at 77 K. These attributes make it widely applicable in the aerospace industry [1,2,3,4,5,6]. Despite these advantages, Ti64 alloy presents significant challenges, including difficulties in machining, high raw material costs, and the necessity for complex forming and processing techniques [7]. Traditional manufacturing methods such as forging are often associated with substantial material wastage, exemplified by a buy-to-fly (BTF) ratio of approximately 25:1 [8].

To this end, wire laser additive manufacturing (WLAM) technology is gaining significant attention [1,9,10]. In additive manufacturing (AM), the use of wire instead of powder generally results in lower material contamination and offers a cost-effective alternative due to the reduced material costs and higher deposition rates [9,11]. It is anticipated that employing WLAM technology for the direct AM of Ti64 components, such as spherical gas tanks for launch vehicles, will result in considerable cost savings by mitigating material waste and reducing the high machining costs inherent in Ti64’s machinability challenges.

Mechanical reliability is crucial for ensuring the structural stability of Ti64 deposits produced via WLAM. Previous research showed that with optimized wire feeding-based AM, the resulting deposits exhibit mechanical properties that are comparable, or even superior, to those of cast and forged Ti64 materials [7,9,10,12,13,14]. However, insufficient process optimization can lead to defects in the deposits, including surface imperfections like spatter and humping, as well as internal flaws such as lack of fusion (LOF) and gas porosity [10,13,14,15,16,17,18,19]. These defects can compromise the mechanical integrity of the material; for example, LOF typically manifests perpendicular to the building direction, adversely affecting fracture toughness and ductility [20,21]. Gas pores, formed by gas entrapment during solidification, contribute to a decrease in tensile strength as their volume fraction increases [16,17]. Therefore, controlling these defects is crucial for maintaining the material’s stability and performance. Non-destructive testing (NDT) is commonly performed to detect internal and surface defects in additively manufactured and welded components. Among these methods, ultrasonic testing (UT) utilizes high-frequency sound waves that propagate through the material to identify internal defects. Additionally, X-ray and computed tomography (CT) inspections employ X-rays or gamma rays to visualize internal defects in 2D or 3D, providing a detailed structural analysis [22].

The microstructure of Ti64 alloy fabricated via wire-based AM varies significantly depending on the thermal history [9,23,24]. Key microstructural features include α colony structures, basket-weave patterns, and martensitic phases. During multi-layer deposition in the WLAM process, the initial β phase, formed at the onset of solidification, is finely structured in an equiaxed shape near the base metal due to the high cooling rates. This equiaxed β phase serves as a nucleation site for subsequent layers, resulting in a coarser columnar grain structure as the deposition height increases [14,23]. Typically, as molten metal solidifies, crystals grow in the direction of the steepest temperature gradient, which is perpendicular to the solid–liquid interface. In the case of the β phase, with its BCC crystal structure, the preferred growth direction is along the <100> axis, leading to competitive grain growth and a reduction in the number of β grains as solidification progresses with increased deposition height [25]. Furthermore, depending on the cooling rate, whether slower or faster than the critical 20 °C/s threshold, different microstructures such as α-colony structures, partial martensite, or complete martensite can form during multi-layer deposition [9,17,24,26]. The accumulated heat from subsequent layer deposition and the re-melting of previous layers result in varying microstructures throughout the deposit: α-colony and Widmanstätten structures near the base, basket-weave structures in the middle, and fine basket-weave and martensitic structures at the top [23].

Mechanically, Ti64 alloy produced by the WLAM process has demonstrated yield strength and ultimate tensile strength values that meet or exceed those of cast and forged counterparts. The yield strength and ultimate tensile strength of Ti64 in cast and forged conditions are 785 MPa and 830 MPa, respectively, with ultimate tensile strength standards being 880 MPa and 900 MPa or higher [7]. Typically, the faster cooling rates in wire-fed AM processes result in finer grain sizes, thus enhancing strength but reducing elongation. However, this reduced ductility can be mitigated through heat treatment above 800 °C [9,25]. Additionally, Ti64 alloys produced via the WLAM process exhibit mechanical anisotropy, primarily due to the columnar grain growth of prior β grains along the building direction. In tensile tests, when the tensile axis is aligned with the building direction, the material shows greater elongation due to significant plastic deformation, as dislocation movement within the grains is facilitated. Conversely, in the side direction, the dislocations encounter grain boundaries, impeding plastic deformation and resulting in higher strength [23]. Fractographic analysis reveals coarse dimple structures on building-direction fracture surfaces and quasi-brittle regions with finer dimples on side-direction fracture surfaces. Chen et al. [7] observed that aligning the wire feeding angle with the heat source reduces mechanical anisotropy by promoting finer, equiaxed grain formation. Therefore, optimizing the process parameters and controlling the microstructure are critical for improving the mechanical properties of the deposits.

When fabricating spherical gas cylinders and tanks using the AM process, post-processing is necessary to address the uneven surface roughness. Additionally, heat treatment is essential for reducing mechanical anisotropy in Ti64 components produced via AM. However, manufacturing a perfect spherical shape directly through AM poses challenges for internal surface treatment and heat treatment processes. Thus, a more efficient approach involves fabricating two hemispheres under identical process conditions, followed by post-processing, heat treatment, and subsequent joining to form the final product. Ensuring the reliability of the joints, particularly concerning defects, microstructure, and mechanical properties, is crucial. While extensive research has been conducted on the joining of Ti64 forged materials using various methods such as arc, laser, and E-beam welding [16,27], studies on the joining of substrates produced by the powder bed fusion (PBF) process using lasers have also been reported [28,29,30].

Typical defects encountered during the laser joining of Ti64 include surface spatter in forged materials, leading to stress concentrations and underfill formation, as well as internal defects like pores, which adversely affect mechanical properties depending on their prevalence. For additively manufactured materials produced via the PBF method, defects such as craters, cold cracks, and gas-induced pores between the fusion zone (FZ) and heat-affected zone (HAZ) have been reported [30,31]. In particular, internal defects are closely related to heat input. As heat input increases, the width and depth of the melt pool expand, and excessive heat input can lead to the formation of porosity in the FZ. Conversely, when heat input is too low, insufficient energy may result in incomplete melting of the filler metal, leading to LOF [32]. These defects significantly impact the mechanical properties of the joints, necessitating mitigation through pre-heating, post-weld heat treatment (PWHT), or optimized processing conditions.

The microstructure of Ti64 alloy during laser joining is process dependent, with variations observed between the base metal (BM), HAZ, and FZ due to differences in cooling rates and thermal gradients. In forged materials, the BM typically exhibits an α + β bimodal structure, while the HAZ, located near the FZ, forms finer acicular α′ or α-lath structures due to faster cooling rates. The FZ typically comprises martensite formed by rapid cooling above the β-transus temperature (980 °C) [27]. In contrast, when joining Ti64 produced by the PBF method, the microstructure in the FZ primarily consists of acicular martensite and fine equiaxed grains within a columnar prior β grain matrix, while the HAZ displays fine equiaxed grains and a basket-weave structure [31]. The FZ tends to be larger in AM-produced materials due to their higher thermal conductivity compared to forged materials [28,31].

The mechanical properties of the weld joints are intricately linked to the microstructure and defects in the joint regions (FZ, HAZ). Omoniyi et al. [31] reported that in laser-joined Ti64 forged materials, the FZ exhibited high microhardness values ranging from 420 to 438 HV due to fine martensite formation, while the HAZ displayed microhardness values of 371 to 392 HV, attributed to coarser martensite. In contrast, Ti64 produced via the PBF method showed FZ hardness values between 416 and 459 HV and HAZ values from 339 to 376 HV, demonstrating that there was no significant difference in hardness between forged and additively manufactured joints. The tensile strength of the joints revealed that specimens produced by the PBF method possessed superior ultimate tensile strength and yield strength compared to forged materials, albeit with lower elongation due to the formation of finer grains under faster cooling conditions [29,30]. The fracture analysis indicated that failure typically occurred in the base material rather than the joint area, with brittle fractures observed in additively manufactured materials and ductile fractures in forged materials [29].

As previously mentioned, studies on the welded joints of forged and PBF-manufactured Ti64 alloys covering defects, microstructure, and mechanical properties have been widely reported. However, to efficiently produce spherical gas bombes or tanks, the approach of building hemispherical sections via additive processes, followed by post-processing, heat treatment, and TIG welding to finalize the product, has recently drawn particular interest to WLAM, especially in terms of material cost and deposition rate. Despite this potential, the TIG welding of WLAM-fabricated Ti64 components remains unreported. Therefore, this study focuses on WLAM-produced Ti64 and its TIG-welded joints, presenting a multifaceted evaluation of defects, microstructure, and mechanical properties to assess overall reliability. As this constitutes the first known investigation of its kind, it is deemed essential groundwork for the eventual application of WLAM-based Ti64 structures in the aerospace industry.

## 2. Materials and Methods

### 2.1. Wire and Substrate Composition

The wires used for AM and TIG welding were supplied by meltio 3D (Barcelona, Spain) and TIPRO International Co. (Xi’an, China), respectively. The substrate was provided by Titanium Metals Corporation (Morgantown, PA, USA). The chemical composition of each material is listed in Table 1, as provided by the supplier. The wire used in both AM processes had a diameter of 1.0 mm, while the TIG welding wire had a diameter of 1.6 mm.

### 2.2. WLAM Process Condition

**Figure 1:**
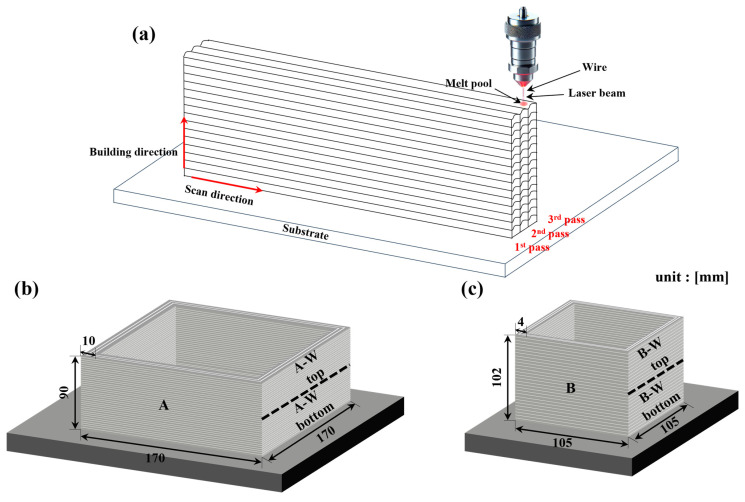
Schematic of the WLAM process. (**a**) WLAM process, (**b**) image of sample A, (**c**) image of sample B.

Since this study was conducted as an industrial project requiring the consideration of inter-machine effects, the WLAM process was carried out using two different machines under separate conditions, as summarized in Table 2. Additive manufacturing was performed with a Coax Printer (Precitec, Gaggenau, Germany) and a Meltio Engine (Meltio 3D, Barcelona, Spain) equipped with a wire laser DED head using an IR laser, and as shown in Figure 1a, each layer’s scan pattern was produced in three passes. To determine the optimal process conditions, a parametric study was conducted by fabricating single-bead and multi-layer coupon specimens. These specimens were sectioned and analyzed using optical microscopy to evaluate cross-sectional density and defect formation. The process parameters that resulted in the highest relative density and minimal defects were selected for this study. The samples were fabricated in a unidirectional manner along the scan direction, resulting in as-built rectangular columns with dimensions of 170 mm × 170 mm × 90 mm × 10 mm and 105 mm × 105 mm × 102 mm × 4 mm (width × length × height × thickness), as illustrated in Figure 1b,c. Subsequently, for TIG welding and to extract specimens for microstructural and mechanical properties analysis, the samples were cut from the base material, surface machined, and further sectioned to the sizes indicated by the dotted lines in Figure 1b,c. TIG welding was performed on A-W top, A-W bottom, B-W top, and B-W bottom. To ensure optimal welding conditions, the edges were machined into a V-groove shape.

### 2.3. TIG Welding Process Condition

**Figure 2:**
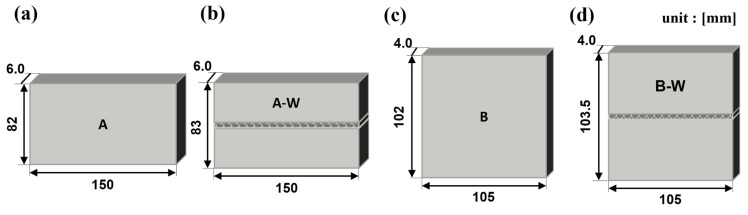
Schematic of samples A and B after surface processing. (**a**) A, (**b**) A-W, (**c**) B, (**d**) B-W.

For TIG welding, an experimental optimization approach was employed to determine the optimal welding conditions. Welding trials were conducted under varying parameters, and the resulting welds were analyzed through cross-sectional observation to assess fusion uniformity and porosity. Based on this evaluation, the parameters that produced the most consistent weld quality with minimal defects were selected. The finalized TIG welding conditions included a current range of 72 to 110 A, a voltage range of 12 to 16 V, and an argon shielding gas flow rate of 12 to 17 L/min. The post-welding specimen conditions are illustrated schematically in Figure 2a–d.

### 2.4. Material Characterization

**Figure 3:**
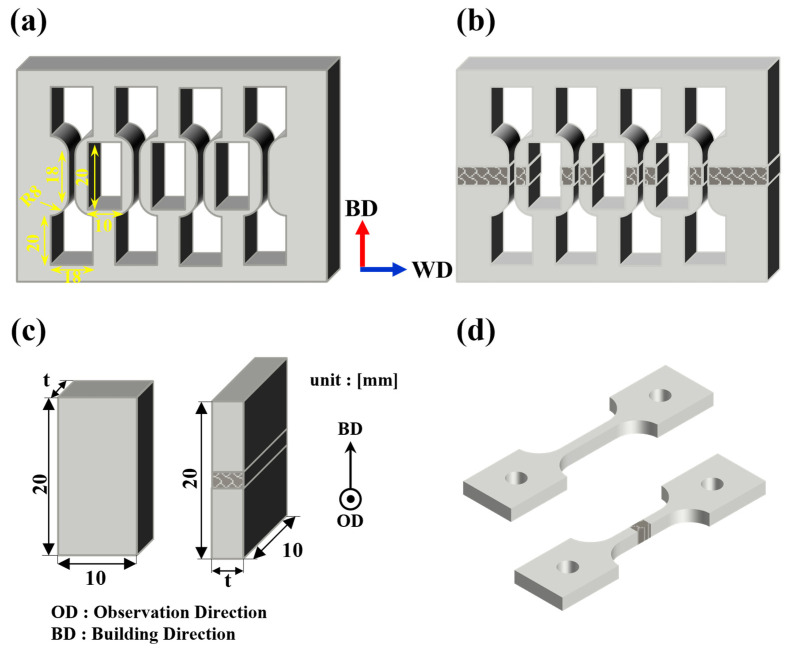
Schematic of a machined sample. (**a**) and (**b**) Image of machined deposit and welded samples, (**c**) microstructure analysis sample image, (**d**) tensile sample image.

To inspect internal defects in the deposited and welded materials, non-destructive testing was conducted using 3D X-ray computed tomography (X-ray CT, XTH320, Nikon, Tring, UK) at the Ulsan Technology Commercialization Center of the Korea Institute of Industrial Technology (KITECH). The results were processed and analyzed with myVGL software (2023.4v, Visual Graphics, Heidelberg, Germany). Following the CT scans, samples were prepared for microstructural analysis and tensile testing. The deposits were sectioned using electrical discharge machining (EDM), and the specimens were prepared according to the dimensions specified in Figure 3a. As depicted in Figure 3a, both the front of the layered material and the side of the welded material were subjected to analysis. To analyze the microstructure of the deposited and welded materials, the processed samples were mounted and polished. An etchant consisting of a mixed solution of 75 mL H2O, 15 mL HNO3, and 10 mL HF was used. The samples were immersed in the etchant for 1 to 2 s, followed by ultrasonic cleaning with ethanol for 20 min. The microstructure was observed using optical microscopy (OM, BX53MRF-S, Olympus, Tokyo, Japan), electron backscattered diffraction (EBSD, EDAX Clarity Super, Ametek EDAX, Berwyn, IL, USA), and energy dispersive X-ray spectroscopy (EDS, X-MaxN, Oxford Instruments, Abingdon, UK). The analysis was conducted using TSL OIM (software, Ametek EDAX, Berwyn, IL, USA), Aztec (software, Oxford Instruments, Abingdon, UK), and Image J (software, ij154-win-java8, National Institutes of Health, Bethesda, MD, USA).

### 2.5. Mechanical Characterization

To assess mechanical properties, tensile tests were conducted using a universal testing machine (TESTCOR-A-TM003/UN300EN-1904131, TESKO Co., Ltd., Jinju, Republic of Korea). The tests were performed at room temperature according to ASTM E8, with a crosshead speed of 0.3 mm/min up to the yield point and 1.1 mm/min beyond the yield point, with each sample tested in duplicate. The tensile specimens were extracted along the building direction, and for the welded materials, the weld zone was centered in the tensile specimen, as illustrated in Figure 3b,d. Yield strength was determined using the 0.2% offset method. Following the tensile tests, the fracture surfaces of the samples were examined using a scanning electron microscope (SEM, IT700, JEOL, Akishima, Japan) at the Korea Institute of Materials Science (KIMS).

## 3. Results

### 3.1. Microstructure Observation

In this study, to compare the microstructures of Ti64 deposits fabricated by WLAM and their TIG weld joints, analysis were conducted using OM, SEM, EBSD, and EDS. The findings are as follows.

**Figure 4:**
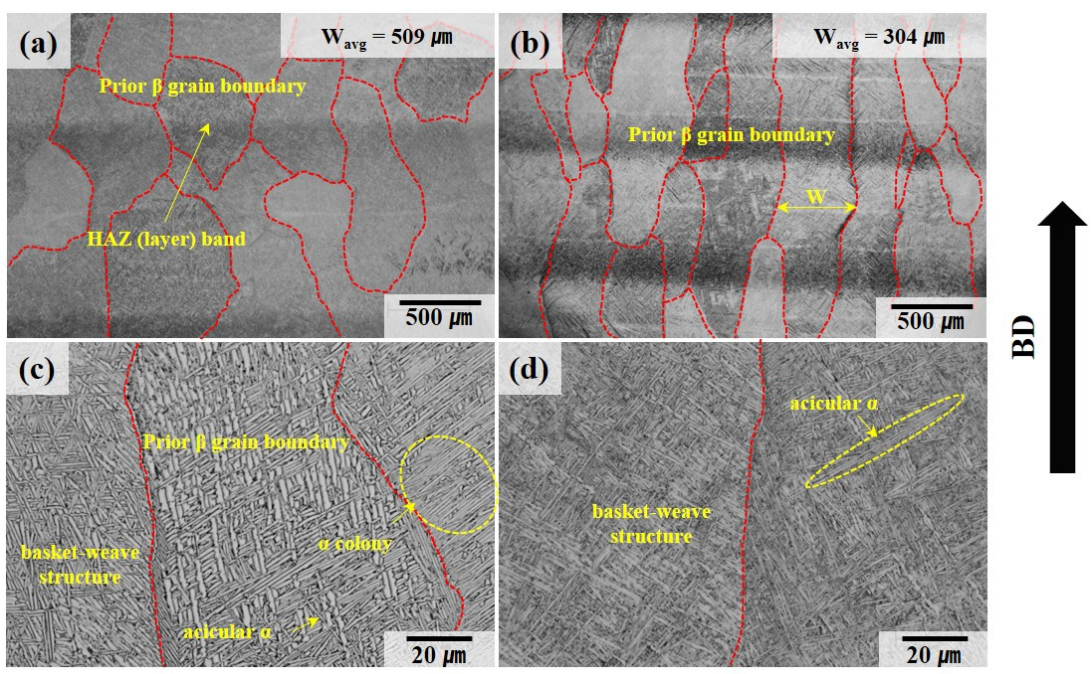
Optical metallographic images of deposit sample A (**a**,**c**) and deposit sample B (**b**,**d**). The red dotted line represents the prior β grain boundary.

The microstructure of the Ti64 alloy fabricated by WLAM is shown in Figure 4. As shown in Figure 4a,b, the prior β grain boundaries, highlighted in red, consist of columnar grains formed through epitaxial growth parallel to the build direction, which corresponds to the direction of the steepest temperature gradient. Figure 4c,d shows that within the prior β grain boundaries, a basket-weave structure composed of a lath α phase is present. Additionally, α colonies growing in a singular direction can be observed adjacent to the prior β grain boundaries [33,34]. Generally, during cooling, when the β phase transforms, a lath α phase with a plate-like morphology forms if the diffusion of alloying elements is adequate. Conversely, an acicular α phase with a non-uniform needle-like shape forms when the diffusion of elements is restricted. Moreover, HAZ bands are visible in the as-built state, consistent with the observations reported by B. Baufeld et al. [9,26,34,35]. However, under conditions of high scan speed or low energy density, the reduced heat dissipation time results in a smaller melt pool volume and a higher temperature gradient, leading to faster cooling rates and narrower prior β grain boundaries [36,37]. Consequently, as indicated by the differences in process parameters shown in Table 2, the average width of the prior β grain boundaries in sample B, which is expected to experience a faster cooling rate, is 304 ± 99 μm, significantly narrower than that in sample A (509 ± 78 μm). Furthermore, the lath α and acicular α phases within the grain boundaries in sample B are more densely packed due to the reduced time available for growth compared to sample A.

**Figure 5:**
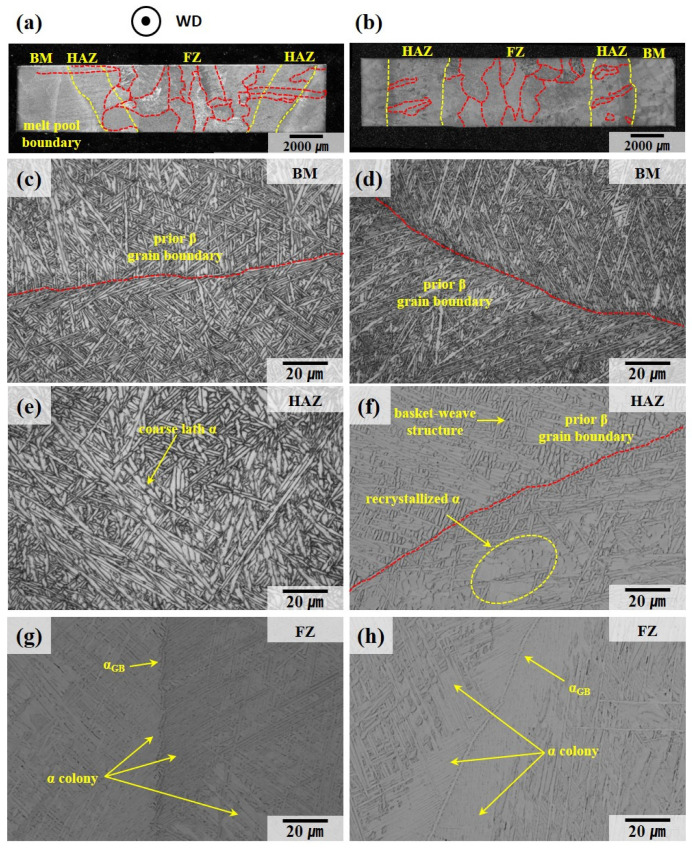
Optical metallographic image of welded specimens: (**a**) low-magnification image of sample A-W, (**b**) low-magnification image of sample B-W, (**c**) base metal in A-W, (**d**) base metal in B-W, (**e**) heat-affected zone in A-W, (**f**) heat-affected zone in B-W, (**g**) fusion zone in A-W, (**h**) fusion zone in B-W. The red dotted line represents the prior β grain boundary.

The microstructure of the welded material, as depicted in Figure 5a,b, can be categorized into three distinct regions: the base metal (BM), which displays the melt pool boundary formed during deposition; the HAZ, characterized by a narrow prior β grain boundary width; and the FZ, which exhibits a wider prior β grain boundary width. As shown in Figure 5e–h, the prior β grain boundaries within the FZ and HAZ present a basket-weave structure and colony structure in the α phase, similar to those observed in the deposited material. It is evident that the width of the lath α phase varies across different regions. Within the HAZ, as depicted in Figure 5e, the lath α phase formed during welding can undergo coarsening due to the thermal effects. Additionally, as illustrated in Figure 5f, recrystallization can lead to the formation of equiaxed α phases. According to the study by Y. Xie et al. [38], regions where the lath α of the basket-weave structure intersect can serve as nucleation sites for recrystallization due to the high density of misfit dislocations. Consequently, at the boundaries of the basket-weave structures located within the HAZ, thermal influence can result in both coarsening and recrystallization.

**Figure 6:**
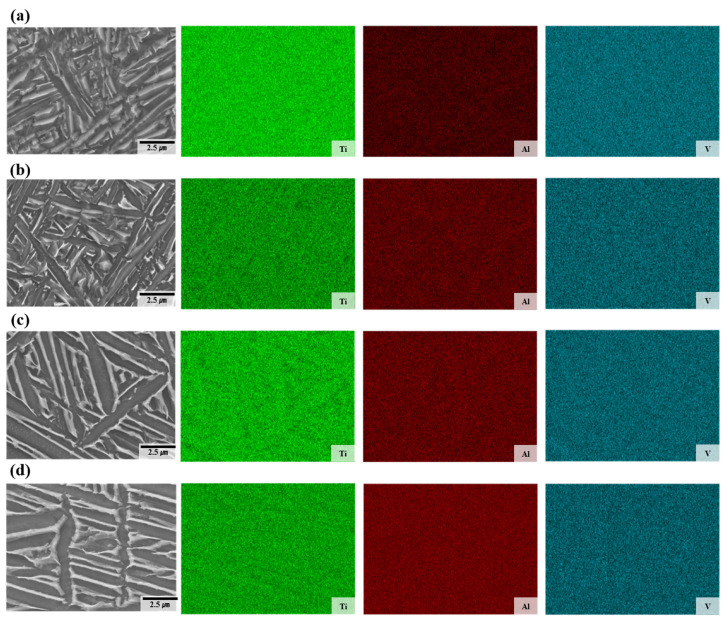
Energy dispersive X-ray spectroscopy maps of the deposits: (**a**) sample A, (**b**) sample B, (**c**) fusion zone in A-W, (**d**) fusion zone in B-W.

Figure 6 shows the distribution of Ti, Al, and V elements in the deposited and welded materials. SEM images also reveal a difference in the width of the lath α phase between the deposited and welded materials, which can be attributed to the rapid cooling characteristic of the WLAM process. This rapid cooling limits the diffusion and partitioning of alloying elements, leading to a uniform elemental distribution, as confirmed by the energy dispersive X-ray spectroscopy (EDS) measurement results. No feasible chemical heterogeneity was observed in any sample in this study, indicating that the rapid cooling in the WLAM process promotes a homogeneous distribution of Ti, Al, and V elements.

**Figure 7:**
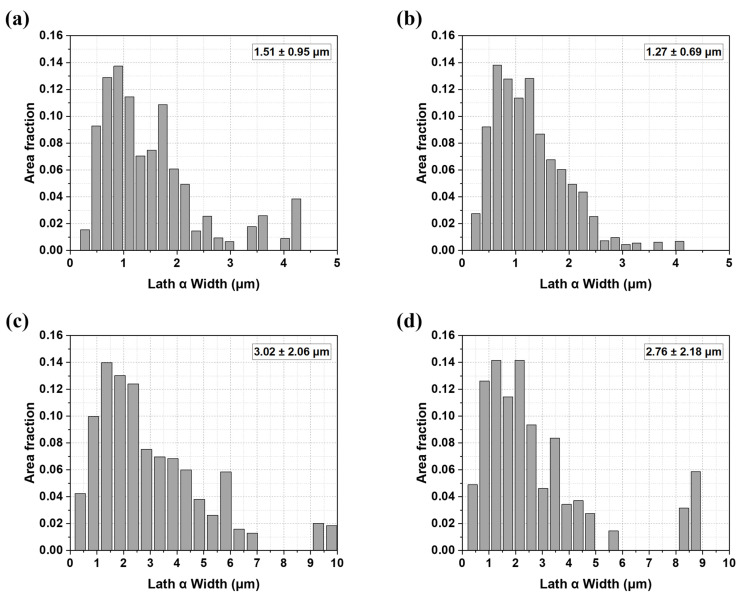
Lath α width distribution of the deposit and the weldment of (**a**) sample A, (**b**) sample B, (**c**) sample A-W, (**d**) sample B-W.

Figure 7 illustrates the width and average value of the lath α phase measured for each specimen. As shown in Figure 7a–d, the average width of the lath α phase varies among the deposit and welded materials: the average widths for the A, B, A-W, and B-W specimens are 1.50 ± 0.95 μm, 1.27 ± 0.69 μm, 3.02 ± 2.06 μm, and 2.76 ± 2.18 μm, respectively.

**Figure 8:**
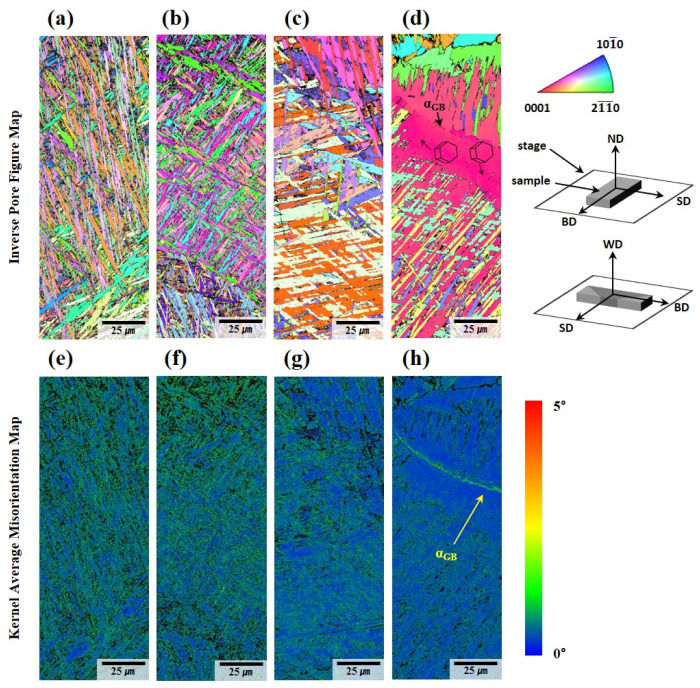
Microstructure of the Ti64 sample analyzed by EBSD: (**a**) IPF map of A, (**b**) IPF map of B, (**c**) IPF map of the fusion zone in A-W, (**d**) IPF map of the fusion zone in B-W, (**e**) KAM map of A, (**f**) KAM map of B, (**g**) KAM map of the fusion zone in A-W, (**h**) KAM map of the fusion zone in B-W.

Figure 8 presents inverse pole figure maps and kernel average misorientation (KAM) maps obtained from electron EBSD measurements. The KAM map, as depicted in Figure 8e–h, reveals variations in geometrically necessary dislocation densities between the deposited and welded materials. During the solidification process, rapid cooling rates impede the diffusion of atoms to equilibrium lattice positions, resulting in significant lattice disorder and elevated elastic strain, thereby introducing misfit dislocations. This phenomenon is amplified in laser-deposited materials due to their inherently higher cooling rates compared to arc-welded materials, where thermal gradients are relatively moderate.

Phase map analysis confirms that the residual β-phase fraction in both the WLAM-deposited and welded regions is exceedingly low—less than 0.006%. This is particularly noteworthy when compared to the typical bimodal α + β microstructure present in cast and forged Ti64 at room temperature, where a higher β-phase fraction is known to enhance ductility. The primary reason for this diminished β-phase content is the rapid cooling inherent to WLAM and TIG welding processes. During these processes, molten pools undergo brief, localized heating followed by rapid cooling, which leads to limited partitioning of alloying elements—particularly β stabilizers like vanadium—and consequently hinders the formation of the β-phase.

In Figure 8d,h, a continuous colony structure with a strong crystallographic texture is prominently observed along the prior β grain boundary. The average width of the acicular α within these colonies is measured to be 1.2 ± 0.26 μm, indicating a fine yet distinct microstructural morphology. These α colonies are believed to nucleate heterogeneously at the prior β grain boundary, where the localized thermal gradients and compositional variations provide favorable conditions for phase transformation. As these colonies grow, their progression halts upon encountering the adjacent basket-weave structure, creating a well-defined microstructural interface. This interaction between the colony α and the basket-weave microstructure underscores the influence of growth kinetics and competing morphological stability on the resultant grain boundary structures.

The grain boundary α_GB_ phase observed along the prior β grain boundary in Figure 8h emerges as a key microstructural feature with significant mechanical implications. This α_GB_ phase acts as a preferential site for crack initiation and propagation during mechanical deformation. Crack activity along α_GB_ is exacerbated by the sharp morphological contrast and inherent brittleness of the α_GB_ phase, contributing to intergranular fracture mechanisms that severely reduce ductility [39].

Using Image J (software, ij154-win-java8), the volume fraction of the α colony structure was measured under each condition, with the following results: specimen A exhibited 6.87 ± 3.62 vol.%; A-W showed 20.59 ± 1.73 vol.%; and specimens B and B-W measured 2.54 ± 0.69 vol.% and 28.26 ± 8.19 vol.%, respectively. These results indicate that the volume fraction of the α colony structure varied according to the cooling rate.

### 3.2. Mechanical Properties Comparison

In order to evaluate the mechanical properties of Ti64 deposits fabricated by WLAM and their TIG weld joints, room-temperature tensile tests were conducted. The results are as follows.

**Figure 9:**
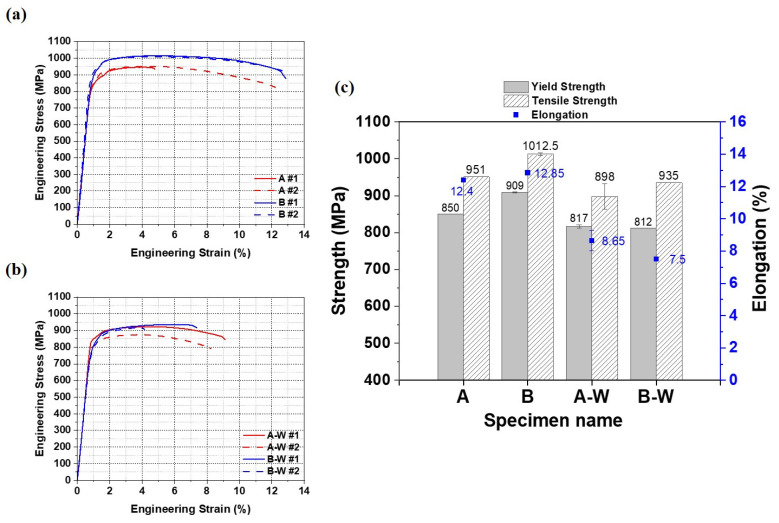
Results of room-temperature tensile tests: (**a**) stress–strain curves of deposits A and B, (**b**) stress–strain curves of welded materials A-W and B-W, (**c**) comparison of tensile properties for deposit and welded materials.

Figure 9 shows the engineering strain–stress curves of the deposited and welded materials, and Table 3 presents the specific values of the tensile test results. The tensile properties of deposits produced via the WLAM process were found to be comparable to or even superior to those of Ti64 produced by WLAM or other laser-based welding methods, as reported in both earlier and more recent studies [7,40,41,42,43]. Both welded specimens, A-W and B-W, exhibited fractures within the weld zone. Comparing A and B, the elongation is similar, but the yield strength and tensile strength differ, with A showing 808 MPa and 945 MPa and B showing 909 ± 1.41 MPa and 1012.5 ± 3.54 MPa, respectively. This difference is likely due to the effects of different cooling rates caused by variations in processing conditions, resulting in narrower prior β grain boundaries (A = 509 ± 78 μm, B = 304 ± 99 μm) and lath α widths (A = 1.50 ± 0.95 μm, B = 1.27 ± 0.69 μm) in sample B, which likely led to higher strength due to the Hall–Petch relationship. When comparing the deposits (A, B) and the welded materials (A-W, B-W), the deposits exhibit superior strength and elongation. The differences in mechanical properties are attributed to differences in microstructure, which will be discussed in more detail in the discussion section. Meanwhile, it can be observed that A #1 in Figure 9a and B-W #2 in Figure 9b have lower elongation compared to other samples (A #1 = 5.15%, B-W #2 = 4.2%). This is likely due to internal defects formed during the AM and welding processes, which led to early failure.

**Figure 10:**
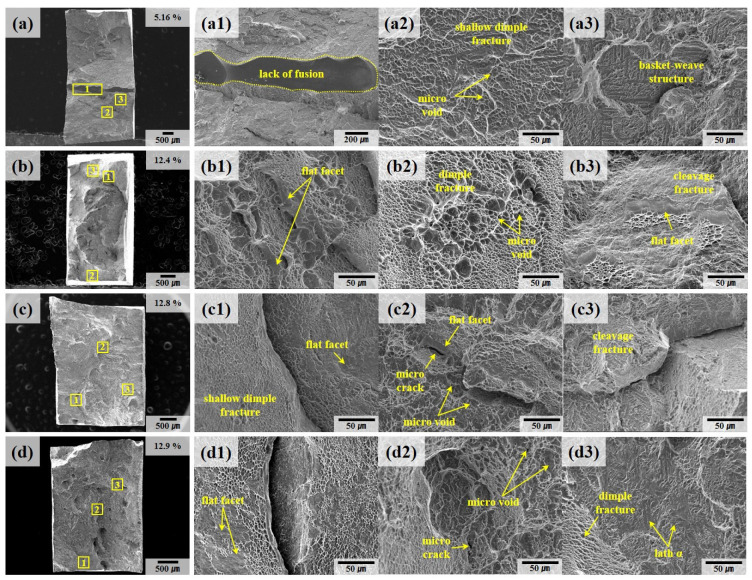
SEM fractography of the deposit specimen after tensile testing: (**a**) A #1, (**b**) A #2, (**c**) B #1, (**d**) B #2. The numbers 1, 2, and 3 represent the regions indicated by yellow rectangular boxes in the low-magnification images.

Figure 10 presents the fracture surfaces of the deposit samples (A, B) analyzed using SEM. Notable, the A #1 specimen, shown in Figure 10a, exhibited an elongation of 5.16%. This relatively low elongation can be attributed to rapid fracture following yielding, driven by the presence of internal defects formed during the deposition process [18]. These defects induce work hardening, coupled with localized stress concentration, thereby reducing the material’s ductility. As highlighted in Figure 10a and the magnified region in Figure 10(a1), a significant defect, characterized by a lack of fusion, is observed traversing the central portion of the fracture surface. Lack of fusion is a defect typically aligned perpendicular to the building direction and, consequently, perpendicular to the applied load direction during tensile testing. This orientation exacerbates the stress concentration at the defect interface during deformation, promoting a Mode I opening fracture. The presence of such a defect compromises the material’s structural integrity and accelerates failure under mechanical loading.

**Figure 11:**
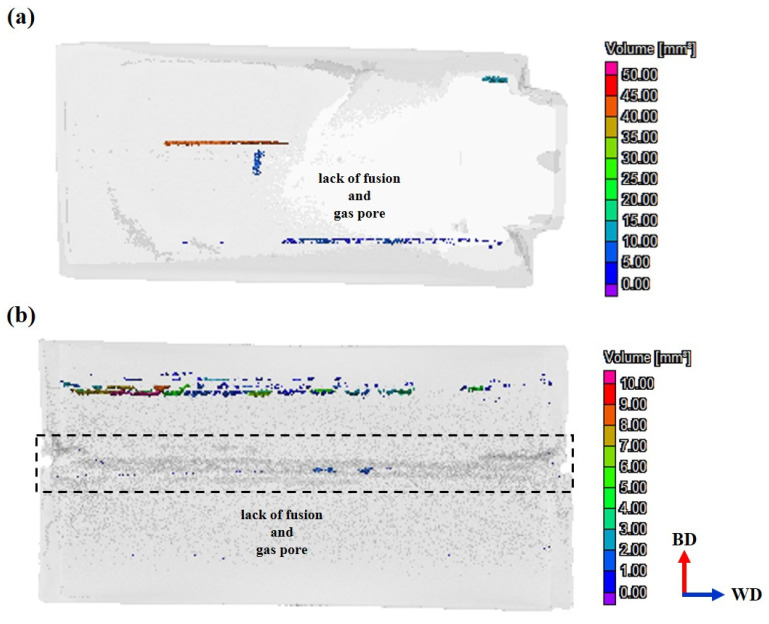
Image of 3D X-ray CT scan analysis: (**a**) sample A, (**b**) sample A-W.

This observation is further substantiated by X-ray CT analysis, as illustrated in Figure 11. Figure 11a displays CT results for specimen A following the WLAM process. The scan reveals internal defects consistent with a lack of fusion, extending along the scan direction, alongside gas pores. These defects are quantified, with an average defect volume of 3.57 ± 7.41 mm^3^, corroborating the SEM findings from Figure 10a. In Figure 11b, CT analysis of specimen A-W (post-WLAM and TIG welding) reveals the presence of defects not only in the base material (deposits) but also in the FZ. Within the FZ, larger porosities were identified with volumes of 1.32 mm^3^, 1.6 mm^3^, and 1.91 mm^3^, alongside smaller porosities with an average volume of 0.04 ± 0.03 mm^3^. These porosities are distributed throughout the FZ and are indicative of the challenges inherent in the deposition and welding processes. The presence of these defects acts as localized stress concentration points during mechanical deformation, which, depending on their volume and distribution fraction, lead to a marked reduction in the material’s overall mechanical performance [44,45].

The Ti64 deposits exhibit a combination of dimple structures of various sizes, as seen in Figure 10(b2,c2,d1), along with cleavage regions featuring flat facets, as shown in Figure 10(b3,c3). It is known that dimple areas are regions of ductile fracture caused by the formation, growth, and coalescence of micro voids during plastic deformation and are primarily formed by basal {0001} <$11\bar{2}0$> and prismatic {$10\bar{1}0$} <$11\bar{2}0$> slip systems in Ti64 deposit and welded materials.

**Figure 12:**
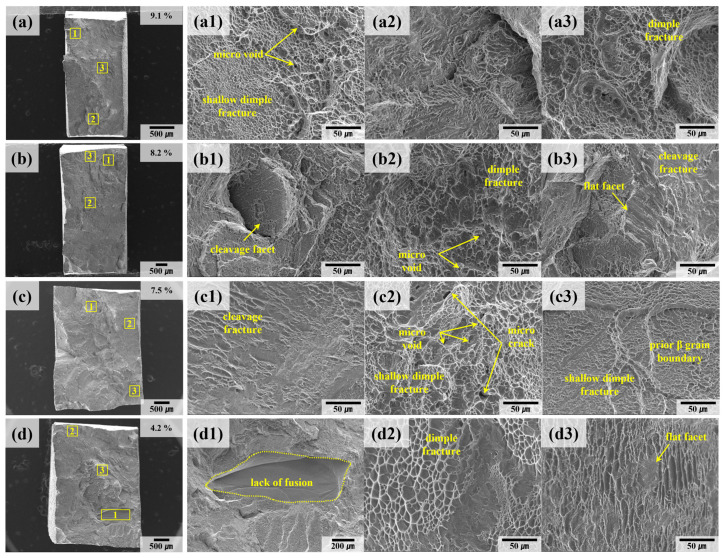
SEM fractography of welded specimens after tensile testing: (**a**) A-W #1, (**b**) A-W #2, (**c**) B-W #1, (**d**) B-W #2. The numbers 1, 2, and 3 represent the regions indicated by yellow rectangular boxes in the low-magnification images.

Figure 12 presents the fracture surfaces of welded samples (A-W, B-W) after tensile testing, providing insights into the failure mechanisms of these materials. In welded specimens, the prior β grain boundaries are oriented perpendicular to the load direction. This orientation facilitates fracture propagation along the grain boundaries, resulting in a flatter fracture surface compared to those observed in the deposited samples. Figure 12(d1) highlights defects analogous to the lack of fusion identified in Figure 10(a1). Although no CT scan data are available for samples B and B-W to conclusively confirm the internal defect distribution, it is inferred that the observed rapid fracture and reduced elongation of 4.2% are primarily due to these surface-visible defects.

The microcracks observed in Figure 10(c2,d2) and Figure 12(c2) originate from the nucleation of micro voids around the lath α phase during plastic deformation. These voids, which form at regions of localized stress concentration, coalesce as deformation progresses, ultimately initiating crack formation and propagation. This coalescence mechanism is particularly detrimental in welded materials, where the thermal gradients and slower cooling rates inherent to the process intensify defect formation [46,47].

Additionally, Figure 10(a2,c1,d1) and Figure 12(a1,c3) reveal the presence of small, shallow dimples on the fracture surfaces of both deposited and welded materials. These dimples, characteristic of ductile fracture, are insufficiently developed, which aligns with findings from prior studies associating this feature with low ductility [23,48,49,50].

The reduced ductility observed in the Ti64 alloy stems from its microstructural characteristics. The high volume fraction of the α phase with its hexagonal close-packed (HCP) structure imposes a high critical resolved shear stress (CRSS) for activating the pyramidal <c + a> slip systems. This high CRSS, in turn, limits dislocation mobility and results in restricted plastic deformation, particularly at room temperature. Consequently, the fracture surfaces exhibit a characteristic quasi-brittle morphology, where dimples indicative of ductile failure coexist with cleavage regions that reflect brittle behavior.

This quasi-brittle fracture mechanism is further influenced by the microstructural anisotropy introduced during the welding process. The slower cooling rates associated with TIG welding promote the formation of coarser grain structures and amplify the presence of α colony phases. These structural features, coupled with the perpendicular orientation of prior β grain boundaries relative to the loading direction, create additional barriers to dislocation movement. As a result, stress concentration at the grain boundaries accelerates crack initiation and propagation, further compromising the material’s elongation and ductility.

In other words, the fracture behavior of the welded Ti64 specimens reflects the intricate interplay between microstructural characteristics, thermal gradients, and loading conditions. The observed defects, grain boundary orientations, and α phase morphology collectively contribute to the material’s quasi-brittle failure mode, highlighting the critical need for process optimization to mitigate such deficiencies.

## 4. Discussion

### 4.1. Microstructural Evolution and Its Impact on Tensile Properties

When comparing the deposited (A, B) and welded materials (A-W, B-W), distinct differences emerge in the width of the lath α phase and the resulting microstructure in the matrix (see Figure 4 and Figure 5). These differences primarily stem from variations in cooling rates caused by the distinct heat sources used in the processes. The β → α phase transformation is heavily influenced by these cooling rates. In laser-based heat sources, the high-speed, concentrated heat supply enables rapid heating and cooling. This leads to rapid solidification and the formation of a dense, fine-grained microstructure. In contrast, arc-based heat sources used in the FZ exhibit slower cooling rates, resulting in broader prior β grain boundaries and thicker lath α phases.

**Figure 13:**
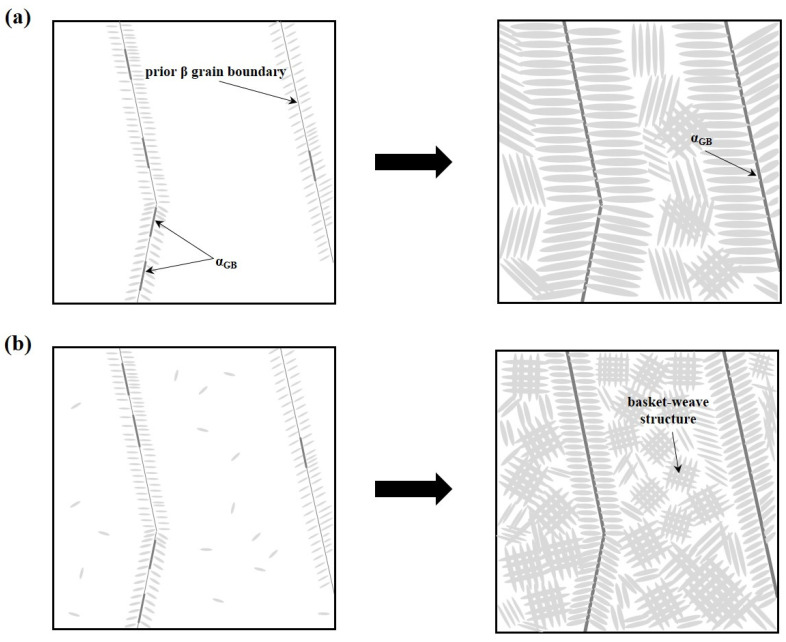
Schematic illustration of α-phase growth according to cooling rate: (**a**) slow cooling rate (welded materials), (**b**) fast cooling rate (deposits).

During the cooling process, α nuclei preferentially form near the prior β grain boundaries, giving rise to grain boundary α (α_GB_) when the material cools below the β-transus temperature (T_β_). Under relatively slow cooling conditions, as illustrated in Figure 13a, the driving force for α nucleation within the grain is diminished. Consequently, coarse α colony structures form through nucleation near α_GB_ or defects where α phases grow in a parallel orientation (see Figure 8d,h). Conversely, rapid cooling, as shown in Figure 13b, enhances the driving force for nucleation, leading to the simultaneous formation of multiple α phases. These phases nucleate not only at the grain boundaries but also within the grains themselves, subsequently growing into plate-shaped lath α structures that intersect to form a basket-weave microstructure (see Figure 8a–b) [51,52,53,54,55,56,57].

The mechanical properties of Ti64 alloy are strongly dictated by its microstructural characteristics. Multiple studies have confirmed that the tensile properties of titanium alloys vary with the width of the prior β grain boundaries and lath α structures [46,58,59,60,61]. This behavior is described quantitatively by the Hall–Petch relationship, as expressed in Equation (1). The relationship demonstrates that narrower lath α structures correlate with higher yield strength [57]. Here, *σ_ys_* represents the material’s yield strength, *σ*₀ is the base yield strength, *k_y_* is the strengthening coefficient, and *s* denotes the lath α width.

(1) $$\sigma_{ys}=\sigma_{0}+k_{y}s^{-1/2}$$

In addition to the Hall–Petch relationship, the tensile strength of Ti64 alloys is significantly influenced by the effective slip length [57]. As the effective slip length decreases, dislocation movement becomes more constrained, leading to enhanced tensile strength. This principle explains why sample B, with a narrower lath α (1.27 ± 0.68 μm), exhibits higher tensile strength than sample A (1.51 ± 0.95 μm) (see Table 3 and Figure 9).

When comparing deposits and welded materials, deposits consistently demonstrate superior strength and elongation. This superiority is attributable to the higher density of misfit dislocations formed during AM compared to welding (see Figure 8e–h). Misfit dislocations are introduced during rapid solidification to alleviate the elastic strain arising from lattice disorder caused by insufficient atomic diffusion time. These dislocations are more prevalent in deposits due to the faster cooling rates, which result in a higher yield strength.

Similarly, deposits feature narrower lath α phases (A = 1.51 ± 0.95 μm, B = 1.27 ± 0.68 μm) compared to the welded materials (A-W = 3.02 ± 2.06 μm, B-W = 2.76 ± 2.18 μm), yielding a shorter effective slip length and, consequently, higher tensile strength (see Figure 7). This correlation underscores the critical role of microstructural refinement in determining mechanical performance.

### 4.2. Ductility Variations in Deposited and Welded Materials

The elongation of the welded materials was observed to be consistently lower than that of the deposits. This disparity in ductility can be attributed to microstructural differences between the two. According to Z. Zhao et al. [57], a high volume fraction of α colony structures in the matrix, combined with thicker lath α phases, significantly reduces ductility. As previously discussed, a rapid cooling rate produces a dense basket-weave structure, while a relatively slower cooling rate promotes the formation of coarse α colony structures.

**Figure 14:**
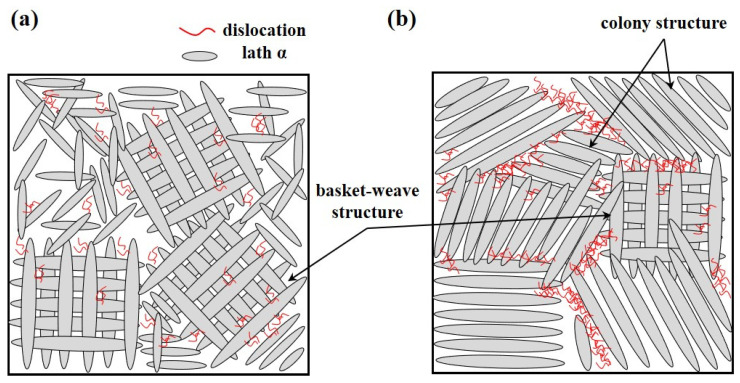
Schematic of dislocation arrangement according to microstructure during deformation: (**a**) dislocation behavior during deformation in a basket-weave structure, (**b**) dislocation behavior during deformation in an α colony structure.

As illustrated in Figure 14a, in cases where the dominant microstructure is a basket-weave structure, dislocations are distributed in multiple directions due to the randomly oriented lath α phases. This dispersion reduces localized stress concentration, improving the material’s ability to accommodate plastic deformation. In contrast, Figure 14b demonstrates that in a colony structure, dislocations tend to move within individual colonies, accumulating at the colony boundaries. This accumulation results in high dislocation density and localized stress concentration, which can rapidly lead to void nucleation, crack propagation, and, ultimately, brittle fracture. In other words, under the same level of plastic deformation, the basket-weave structure distributes dislocations more evenly, whereas the colony structure concentrates dislocations at specific boundaries, increasing the likelihood of fracture. Furthermore, the thick α_GB_ phase, which forms predominantly in the weld region, serves as a preferential site for crack propagation during deformation. These thick α_GB_ layers act as barriers to dislocation motion, exacerbating stress localization and reducing the overall ductility of the material.

**Figure 15:**
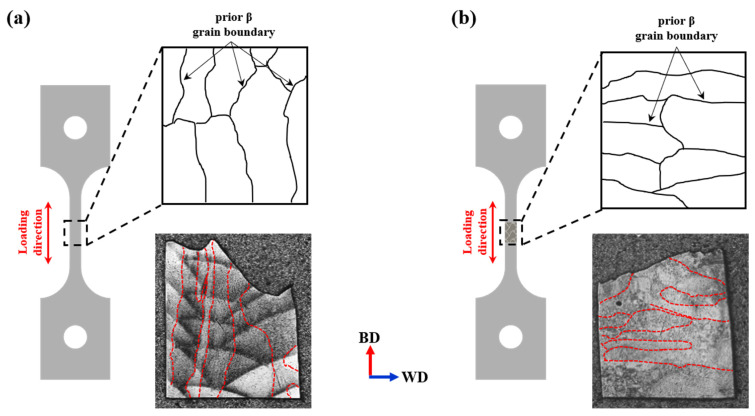
Schematic showing the growth direction of prior β grain boundaries in the tensile specimen: (**a**) image of the deposit specimen, (**b**) image of the welded specimen. The red dotted line represents the prior β grain boundary.

The growth direction of prior β grain boundaries also plays a critical role in determining ductility, as highlighted in the study by B. E. Carroll et al. [62]. The prior β grain boundary typically grows in the direction of the steepest temperature gradient. In deposits, as shown in Figure 15a, the grain boundaries align parallel to the building direction, which coincides with the loading direction during tensile testing. This parallel alignment facilitates dislocation movement along the loading axis, allowing for greater plastic deformation.

In contrast, Figure 15b illustrates that in welded materials, the prior β grain boundaries are oriented perpendicular to the loading direction. This perpendicular alignment impedes dislocation mobility across the grain boundaries, resulting in restricted plastic deformation and reduced ductility due to mechanical anisotropy. These microstructural differences, combined with the slower cooling rates in the weld region, lead to the formation of coarser lath α phases, a significant prevalence of α colony structures, and unfavorable grain boundary orientations. Together, these factors contribute to the inferior ductility observed in welded materials (A-W, B-W) compared to their deposited counterparts.

In the future, research focusing on applying hot isostatic pressing or other appropriate post-processing techniques to induce microstructural homogenization and enhance mechanical properties is required. Furthermore, studies that evaluate long-term reliability through cryogenic property and fatigue tests, as well as those that quantitatively analyze stress concentration and fracture behavior, are also necessary.

## 5. Conclusions

This study examined the microstructural and mechanical properties of WLAM Ti64 alloy following TIG welding. Defect characterization, microstructural analysis, and room-temperature tensile tests were conducted to evaluate the mechanical performance of both as-built and TIG-welded samples, emphasizing the influence of microstructural variations. The key findings are summarized below:

1. Insufficient heat input or a slow wire feed rate during wire-feed AM and TIG welding results in internal defects such as lack of fusion and gas pores. These defects significantly degrade mechanical properties, with their impact proportional to defect size and fraction. Process optimization and post-weld heat treatment are essential to minimize defect formation and improve material performance.
2. The microstructure of the Ti64 alloy varied significantly with cooling rates during the WLAM process. Low heat input and high scan speeds, as in sample B, created steep temperature gradients, leading to faster cooling rates and narrower prior β grain boundaries and lath α phases. However, the elemental partitioning of Ti, Al, and V due to cooling rate differences was not discernible under current analysis conditions.
3. Deposits fabricated using laser heat sources exhibited faster cooling rates than TIG-welded materials, leading to narrower lath α phases and prior β grain boundaries with higher dislocation densities. Consequently, the deposits displayed superior yield strength and tensile strength. In contrast, welded materials showed reduced ductility due to mechanical anisotropy, stemming from the perpendicular orientation of prior β grain boundaries relative to the loading direction and the prevalence of α colony structures. These microstructural characteristics localized dislocation accumulation during deformation, exacerbating stress concentration and reducing plasticity.
4. Both Ti64 deposits and welded materials exhibited quasi-brittle fracture characteristics, marked by a combination of dimples and cleavage regions on the fracture surfaces. While work hardening occurred during plastic deformation, indicating some dislocation activity, the high fraction of α phase with its HCP structure severely restricted overall slip activity, further contributing to the limited elongation and the transition to brittle fracture behavior observed at room temperature.

## Figures and Tables

**Table 1:** Chemical composition of Ti64 wire and substrate used in present work (supplied by the producer).

wt. %	Ti	Al	V	Fe	C	Other
AM wire	Bal.	5.5	3.5	0.4	0.08	0.22
Weld wire	Bal.	6.16	3.97	0.137	0.007	0.16
Substrate	Bal.	6.08	3.67	0.12	0.02	0.20

**Table 2:** Process parameters used in the WLAM process.

WLAM Process Parameter	Sample A	Sample B
Laser beam power (W)	3200	900
Laser scan speed (mm/min)	500	600
Wire feed rate	2.5–3.5 (m/min)	15.3 (mm/s)
Scan path	3	3

**Table 3:** Room-temperature static tensile properties of deposit and welded materials.

	Yield Strength (MPa)	Tensile Strength (MPa)	Elongation (%)
A #1	808	945	5.15
A #2	850	951	12.4
B #1	910	1015	12.8
B #2	908	1010	12.9
A-W #1	820	922	9.1
A-W #2	814	874	8.2
B-W #1	812	935	7.5
B-W #2	804	917	4.2

## Data Availability

Data are contained within the article.
